# Capitalizing Resolving Power of Density Gradient Ultracentrifugation by Freezing and Precisely Slicing Centrifuged Solution: Enabling Identification of Complex Proteins from Mitochondria by Matrix Assisted Laser Desorption/Ionization Time-of-Flight Mass Spectrometry

**DOI:** 10.1155/2016/8183656

**Published:** 2016-09-07

**Authors:** Haiqing Yu, Joann J. Lu, Wei Rao, Shaorong Liu

**Affiliations:** Department of Chemistry and Biochemistry, University of Oklahoma, 101 Stephenson Parkway, Norman, OK 73019, USA

## Abstract

Density gradient centrifugation is widely utilized for various high purity sample preparations, and density gradient ultracentrifugation (DGU) is often used for more resolution-demanding purification of organelles and protein complexes. Accurately locating different isopycnic layers and precisely extracting solutions from these layers play a critical role in achieving high-resolution DGU separations. In this technique note, we develop a DGU procedure by freezing the solution rapidly (but gently) after centrifugation to fix the resolved layers and by slicing the frozen solution to fractionate the sample. Because the thickness of each slice can be controlled to be as thin as 10 micrometers, we retain virtually all the resolution produced by DGU. To demonstrate the effectiveness of this method, we fractionate complex V from HeLa mitochondria using a conventional technique and this freezing-slicing (F-S) method. The comparison indicates that our F-S method can reduce complex V layer thicknesses by ~40%. After fractionation, we analyze complex V proteins directly on a matrix assisted laser desorption/ionization, time-of-flight mass spectrometer. Twelve out of fifteen subunits of complex V are positively identified. Our method provides a practical protocol to identify proteins from complexes, which is useful to investigate biomolecular complexes and pathways in various conditions and cell types.

## 1. Introduction

Density gradient ultracentrifugation (DGU) is a great technique for separating subcellular organelles [1–3], exosomes [4–6], protein complexes [7–9], and so forth on the basis of buoyant density differences. An important feature of DGU is that the resolved components remain very close to their biological states. DGU has also been proven to be an effective tool for purifying nanoparticulate materials [10–12]. Under ultracentrifugation conditions, particles having the same buoyant density travel along the axis of the centrifugal force to form an isopycnic layer. When swing bucks are used, the isopycnic layers are perpendicular to the tube wall. Accurately locating different isopycnic layers and precisely extracting solutions from these layers play a critical role in achieving high-resolution DGU separations.

However, it is challenging to harness all its resolving power because the resolved layers (or bands) can be disturbed/dispersed during solution retrievals. Currently, there are three common protocols for retrieving isopycnic layer solutions. (i) A needle is used to aspirate the gradient solution slowly from the top [13–15]; the needle moves downward as the solution level lowers. (ii) A needle is fixed at the top while a dense solution is introduced to the bottom [16, 17] of a gradient solution to facilitate the solution to be aspirated out via the needle. Alternatively, a needle can be placed at the bottom while an air pressure is introduced to the top to drive the solution out via the needle [18]. (iii) A hole is created at the bottom of the centrifuge tube to allow the gradient solution to flow out (by gravity force) [19, 20]. Apparently, as the solution moves inside the centrifuge tube and as it flows to the needle, the isopycnic bands are broadened.

To eliminate the above band-broadening effect, Cole and Brooks Jr. [21] added some acrylamide in the gradient solution and allowed it to polymerize after isopycnic layers were formed. This method fixed the resolved isopycnic layers, but extracting organelles or protein complexes became troublesome. Mccrea et al. [22] fixed the isopycnic layers by dry ice freezing and fractionated the desired solutions by cutting and thawing. A few years later, Berg and Durand [23] revisited this technique but did not achieve much improved resolutions, possibly due to unoptimized freezing and cutting conditions. More recently in a patent application, Macfarlane et al. [24] mentioned freezing and cutting DGU solution to isolate targeted lipoproteins. Unfortunately, they did not disclose their experimental protocol in detail or explain why they used this technique to retrieve the isopycnic layer solutions. Presumably, other methods broadened the isopycnic layer bandwidths too much.

**Figure 1:**
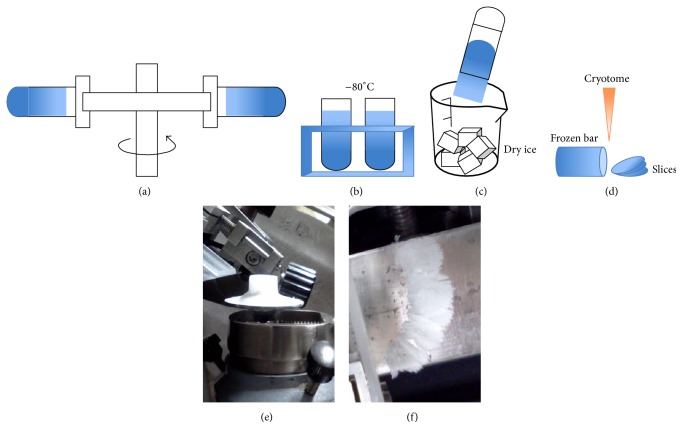
Major steps for F-S method. (a) Sample being centrifuged at 40000 rpm. (b) Gradient sucrose solution being frozen at −80°C; (c) ice bar being unloaded from centrifuge tube into dry ice. (d) Frozen bar being sliced. (e) Frozen bar fixed on stage. (f) Status and shape of freshly sliced ice pieces.

In this technique note, we develop a DGU procedure by freezing the solution rapidly (but gently) after centrifugation to fix the resolved layers and by slicing the frozen solution to fractionate the sample (see Figure 1). Because the thickness of each slice can be controlled to be as thin as 10–40 micrometers, we retain virtually all the resolution produced by DGU. To demonstrate the effectiveness of this method, we fractionate complex V from HeLa cell mitochondria using a conventional technique (aspirating gradient solution slowly from the top) and this F-S slicing method. The comparison indicates that the F-S method is much more effective in retaining DGU resolutions than the conventional technique. Because the sample becomes much less complicated after we isolate mitochondria and separate complex V, we can analyze the proteins directly on a matrix assisted laser desorption/ionization, time-of-flight mass spectrometer (MALDI-TOF-MS). Twelve out of fifteen subunits of complex V are positively identified. Since mass spectrometry (MS) has emerged as the preferred method for in-depth characterization of the protein components of biological systems [25], a practical protocol of sample preparation for MS analysis is essential for studying the composition, regulation, and function of molecular complexes and pathways. Our method can significantly increase sample purity and yield, which may provide a powerful tool for identifying the target biomolecules bundled in various protein complex.

## 2. Experimental

### 2.1. Materials and Reagents

Dulbecco's Modified Eagle's Medium (DMEM) was purchased from Santa Cruz Biotechnology, Inc. (Dallas, TX). Gibco® fetal bovine serum, penicillin-streptomycin (10,000 U/mL), HEPES buffer (1 M), native protein standard, and Novex® HRP Chromogenic Substrate (TMB) were purchased from Life Technologies*™* (Grand Island, NY). Protease inhibitor cocktail (P-2714) was bought from Sigma-Aldrich (Louis, MO). Lauryl maltoside was purchased from VWR International LLC (Radnor, PA). Primary antibody, mouse monoclonal to complex V alpha (ab14748), and secondary antibody, rabbit anti-mouse IgG (HRP) (ab6728), were obtained from Abcam® (Cambridge, MA). Nitrocellulose membrane was bought from Bio-Rad (Hercules, CA). The optimum cutting temperature (OCT) compound was purchased from Ted Pella Inc. (Redding, CA). All other chemicals were purchased from Sigma-Aldrich Corporation (St. Louis, MO).

### 2.2. Buffer Compositions

Several buffer solutions were used in this work. Mitochondria buffer contained 250 mM sucrose, 10 mM HEPES, and 1 mM EDTA (pH 7.0). Phosphate buffered saline (PBS) contained 137 mM NaCl, 2.7 mM KCl, 10 mM Na_2_HPO_4_, and 2 mM KH_2_PO_4_ (pH 7.4). Protease inhibitor cocktail stock solution was prepared by dissolving all the lyophilized powder in the bottle with 10 mL double-deionized water (ddH_2_O). All sucrose solutions were prepared in 10 mM HEPES and 0.05% w/v lauryl maltoside (pH 7.0). Western Blot transfer buffer contained 24 mM Tris, 194 mM glycine, and 20% methanol (pH 8.0). TBST buffer contained 20 mM Tris, 150 mM NaCl, and 0.05% Tween 20 (pH 7.5). In MALDI-TOF-MS experiments, matrix A contained 10 mg/mL *α*-cyano-4-hydroxycinnamic acid (CHCA) in 70% v/v acetonitrile (CAN)-water with 5% v/v formic acid (FA); matrix B contained 10 mg/mL 2.5-dihydroxybenzoic acid (DHB) in 70% v/v ACN-water with 0.05% v/v trifluoroacetic acid (TFA).

### 2.3. Mitochondria Preparation

HeLa cells were cultured in DMEM (Invitrogen, Green Island, NY) supplemented with 10% fetal bovine serum (FBS), penicillin (100 units/mL), and streptomycin (100 units/mL). Cells were incubated in a humidified atmosphere with 5% CO_2_ at 37°C, and about 5 × 10^7^ fresh cells were collected after trypsinization and washed three times with PBS. After washing, cells were suspended in 1 mL PBS with 25 *μ*L protease inhibitor cocktail stocking solution. The cells in PBS were broken with a Branson Sonifier® 450 ultrasonic cell disruptor three times (5 s at a time) on ice. All cell debris and nuclei were removed by centrifugation at 1000 ×g for 10 min at 4°C. Mitochondria were collected from the supernatant by centrifuging at 12,000 ×g [26] for 20 min at 4°C and washed with PBS buffer. The obtained mitochondrial pellets were stored in mitochondrial buffer at −80°C before use.

### 2.4. Major Steps of F-S Method

**Step 1 (centrifugation).**

The mitochondrial pellets were suspended to a concentration of 5.0 mg/mL in a mitochondrial buffer containing 1.0% w/v lauryl maltoside [27]; lauryl maltoside is used to promote mitochondrial membrane solubilisation. This solution was incubated on ice for 30 min. A gradient solution was prepared by, respectively, pipetting 250 *μ*L of 43, 40, 37.5, 35, 32.5, 30, 27.5, 25, 22.5, 20, 17.5, 15, and 10% sucrose (all in 10 mM HEPES, pH 7.0, 0.05% w/v lauryl maltoside) into an Ultra-Clear*™* centrifuge tube (Beckman Coulter, Inc., CA). 0.50 mL of the mitochondrial sample was loaded on above sucrose gradient and centrifuged for 20 h at 40000 rpm in a SW 50.1 rotor on Beckman Optima*™* LE-80 K machine (Beckman Coulter). Theoretically, the F-S method could be applied to separation of other complexes, proteins, particles, and so forth, which could be achieved by density sucrose centrifugation or DGU.

**Step 2 (freezing gradient solution).**

After centrifugation, the centrifuge tube with the sucrose gradient was carefully taken out from the swing bucket and immediately moved into a −80°C freezer for more than 1 h (see Figure 1(b)). The tube should be kept in a vertical orientation until all the solution was frozen. “A frozen gradient solution” was also referred to as “a frozen bar.”

**Step 3 (retrieving frozen bar).**

To take the frozen bar out of the centrifuge tube, the −80°C tube was firstly immerged into room temperature water for 5 s, and then a precooled acetonitrile (−45°C) was added on the top of the tube. Gently press the tube wall with fingers to facilitate the cold acetonitrile to enter the space between the frozen bar and the tube wall. Once the frozen bar got loose, gently unload the frozen bar into a dry ice container (see Figure 1(c)).

**Step 4 (slicing frozen bar).**

The frozen bar was first roughly cut into several pieces (each piece had a thickness of approximately 10 mm) with a regular blade. The CRYO-CUT machine with a model 845 microtome blade (American Optical Corporation Scientific Instrument, Buffalo, NY) was precooled at −30°C. A frozen bar or a roughly cut thick piece was secured to a stage using OCT compound as an adhesive (see Figure 1(e)). The stage was put at −30°C for 15 min to allow the adhesive to solidify. Then, the stage was fixed to a three-way adjustable holder and set an appropriate angle for the blade to slice the sample in a vertical direction. After a desired number of pieces were produced, they were collected into an Eppendorf tube as a fraction. This slicing process was repeated until the frozen bar was exhausted. All fractionated samples were stored in a −80°C refrigerator.

**Step 5 (preparation of sample for analysis).**

A selected portion of the fractionated sample was thawed at 4°C and kept on ice. The salt and sucrose in the sample were removed by an Amicon Ultra-0.5 Centrifugal Filter Unit with Ultracel-3 membrane (UFC500396, Sigma-Aldrich). After the sample was loaded into the filter unit, 400 *μ*L of 0.05% lauryl maltoside was added to dilute the sample and the unit was spanned at 14000 rpm at 4°C on HERMEL Z 36 HK centrifuge (Wehingen, Germany) to remove the solvent. This washing procedure was repeated three times, each with 400 *μ*L of 0.05% lauryl maltoside. Finally, protein dissolved with 15 *μ*L of 0.05% lauryl maltoside. The total protein recovery was greater than 90%. For gel electrophoresis, the protein concentration of the sample was estimated by BioTeK microreader (Winooski, VT) to be ~1.4 mg/mL.

### 2.5. Blue Native Polyacrylamide Gel Electrophoresis (BN-PAGE)

Blue native gradient gels (4−15% acrylamide) were casted according to published protocols [28, 29] to analyze the fractionated samples. 2 *μ*L concentrated samples (2~3 *μ*g) were loaded on the gel and run the electrophoresis. A silver stain process was employed for staining resolved proteins. A native protein standard was loaded on the gel beside samples to indicate the molecular weight of mitochondrial complexes. The band densities on the gels were estimated by Image Lab 5.0 (Bio-Rad, NY).

### 2.6. Identification of Mitochondrial Complex V by Western Blot

After BN-PAGE, the gel was gently removed from gel cassette to a Western Blot sandwich. Following the electric transfer under 15 V in a transfer buffer for 1 h in a Life Technologies mini gel system, the nitrocellulose membrane was taken out and blocked with 5% milk/TBST for 1 h at room temperature. A primary antibody, mouse monoclonal to complex V alpha (ab14748), and a secondary antibody, rabbit anti-mouse IgG (HRP) (ab6728), were applied on membrane for 1 h at room temperature successively. Then, Novex HRP Chromogenic Substrate (TMB) was added for visualization. The image of the membrane was obtained on a ChemiDoc*™* MP Imaging system (Bio-Rad, CA).

### 2.7. MALDI-TOF-MS Analysis

MS analysis was carried out using an AB Sciex MALDI-TOF-MS (model 4800 plus, Darmstadt, Germany) equipped with a high-mass detector (HM2, CovalX AG, Zurich, Switzerland). Ionization was achieved with N_2_ laser (377 nm). Positively charged ions were analyzed in a linear mode. MALDI matrix was prepared by mixing matrix A with matrix B at a 1 : 1 ratio right before use. Ice slices obtained from our F-S method were thawed on ice and filtered with an Amicon ultra filter column (Sigma-Aldrich, St. Louis, MO) to remove sucrose and salts. After filtration, the sample was suspended into DDI water with 0.05% w/v lauryl maltoside. The sample was mixed with MALDI matrix at a 1 : 1 ratio, and the mixture was then deposited on a MALDI-TOF-MS target for analysis. Mass spectra of proteins were obtained by averaging 1000 random laser shots on each sample without searching for hot spots.

## 3. Results and Discussion

### 3.1. Comparison between Conventional Aspiration Technique and F-S Method

**Figure 2:**
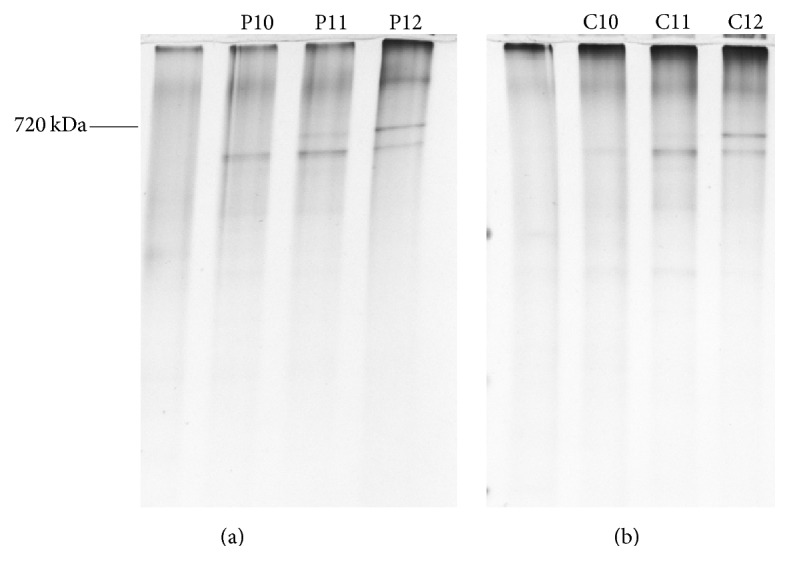
Comparison of distribution of mitochondrial complex V in three adjacent aliquots (sucrose density 34–43%) by BN-PAGE. (a) BN-PAGE results of aliquots fractionated by pipetting method. (b) BN-PAGE results of aliquots fractionated by F-S method.

To examine the difference between a traditional aspiration technique and our F-S method, we prepared two identical mitochondrial samples and loaded them on two 10–43% sucrose gradient solutions. After centrifugation, one tube was immediately frozen at −80°C, and then the frozen bar was roughly cut into 12 even pieces (each with a thickness of 3.5 mm). The solution in another tube was carefully aspirated out using a 50 *μ*L pipette, and the solution was divided into 12 equal aliquots (310 *μ*L/aliquot). These two sets of samples were analyzed by BN-PAGE, and the results are presented in Figure 2. In the samples produced by the aspiration technique, complex V was found in P10, P11, and P12 (aliquots 9, 10, and 11). In the samples produced by our F-S method, although complex V was also found in three aliquots (C10, C11, and C12), it was focused primarily in C11 (piece 11).

To compare the two techniques more closely, we redid the above experiment but with reduced aliquot size. With the F-S method, we sliced the frozen bar into 30 *μ*m thick slices and combined 10 consecutive slices as one fraction. Each fraction was equivalent to a 300 *μ*m thick piece frozen bar or approximately 27 *μ*L of the gradient. With the traditional aspiration technique, we aspirated the gradient and distributed it into 27 *μ*L per fraction. Since we knew roughly where complex V was located, only solutions near that region were taken for BN-PAGE and Western Blot.

**Figure 3:**
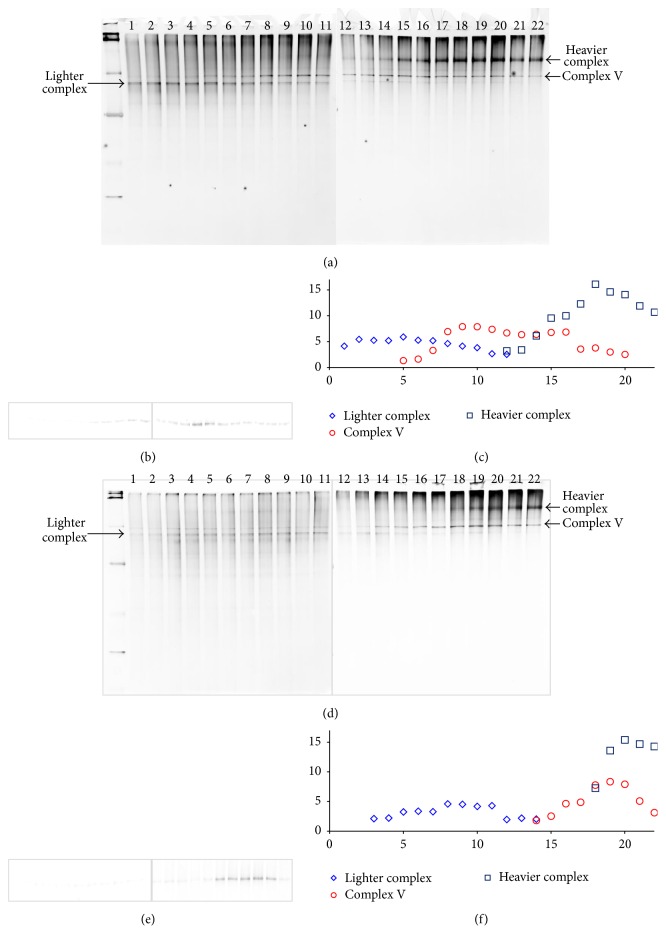
Detailed comparison of protein complex distribution. (a), (b), and (c) BN-PAGE, Western Blot, and distribution profile of samples prepared by conventional aspiration method. (d), (e), and (f) BN-PAGE, Western Blot, and distribution profile of samples prepared by F-S protocol.

Figure 3 presents the results. With the traditional aspiration technique, complex V was spread over 16 fractions (from 5 to 20) (see Figure 3(a)). With our F-S method, complex V was focused in 9 aliquots (from 14 to 22) (see Figure 3(c)); ~40% bandwidth reduction was achieved. The Western Blot results in Figures 3(b) and 3(d) confirmed these observations. As a matter of fact, more focused layers were obtained for the relative lighter and heavier complexes (compare Figures 3(c) and 3(f)).

### 3.2. Analysis of Complex V Proteins by MALDI-TOF-MS

**Figure 4:**
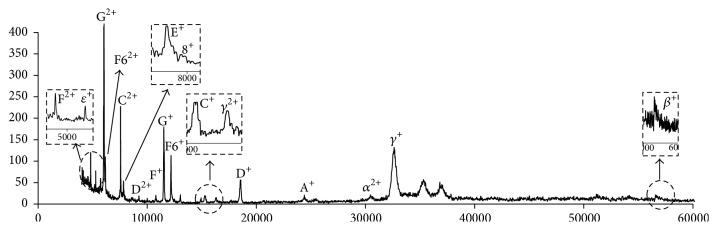
MALDI-TOF-MS of integral protein of mitochondria complex V.

An excellent feature of our method is that, after mitochondria isolation and complex V separation, the sample becomes much less complicated. To exhibit the benefit of this, we analyzed this sample directly on a MALDI-TOF-MS for subunit identifications. We combined two fractions (e.g., p16 and p17 in Figure 3(f)). After sucrose and salts inside the sample were removed by ultra filter column, the proteins were resuspended in 0.05% w/v lauryl maltoside water solution. After the sample was mixed with matrices, it was deposited on a MALDI-TOF-MS plate for target analysis.  Figure 4 presents the MS spectrum; 12 of the 15 subunits of mitochondrial complex V were positively identified (see Table 1).

## 4. Conclusions

We have developed an F-S protocol to capitalize the resolving power of DGU. Compared to a traditional aspiration technique, our method has reduced the complex V bandwidth by a fact of more than 40%. Because the sample has become much less complicated after mitochondria isolation and complex V separation, we have analyzed proteins inside the sample directly utilizing a MALDI-TOF-MS and positively identified 12 out of 15 subunits of complex V. The F-S method has other advantages over other traditional methods. The sliced samples can be fractionated freely; one can use a single slice as a fraction, or one can combine any number of slices into one fraction for analysis. The sliced samples can be stored for a long period of time because they remain frozen all the time. Recent application of DGU for structural sorting of single-walled carbon nanotube (SWCNT) samples has created a need for highly selective extraction of closely spaced layers formed in the centrifuged tube [15]. We are testing our method for SWCNT separations. We are also testing its effectiveness for separating other proteins and complexes. These results will be published elsewhere. However, a drawback of the F-S method is its extended time for sample preparations, compared to traditional aspiration technique.

## Figures and Tables

**Table 1:** Molecular weight of each subunit of complex V^*∗*^.

Subunit	MW	
Alpha (*α*)	59,754	√
Beta (*β*)	56,563	√
Gamma (*γ*)	32,997	√
B	28,910	*✕*
A	24,817	√
OSCP	23,277	*✕*
D	18,360	√
Delta (*δ*)	17,490	*✕*
C	14,277/14638/14693	√
F6	12,588	√
G	11,387	√
F	10,787	√
8 (A6L)	7,992	√
E	7,802	√
Epsilon (*ε*)	5,648	√

^*∗*^Data cited from human mitochondrial protein database was developed by the National Institute of Standards and Technology. √ indicates being identifiable, and *✕* indicates being not identifiable.
